# Community perception and utilization of services for the severe wasted children aged 6–59 months in the Forcibly Displaced Myanmar Nationals and their nearest host communities in Bangladesh: a qualitative exploration

**DOI:** 10.3389/fnut.2024.1235436

**Published:** 2024-02-14

**Authors:** Mahfuzur Rahman, Nurun Nahar Naila, Md. Munirul Islam, Mustafa Mahfuz, Aklima Alam, Gobinda Karmakar, Anjuman Tahmina Ferdous, Muhammad Abu Bakr Siddique, Piyali Mustaphi, Tahmeed Ahmed

**Affiliations:** ^1^School of Health and Rehabilitation Sciences, The University of Queensland, St Lucia, QLD, Australia; ^2^Nutrition Research Division, icddr,b, Dhaka, Bangladesh; ^3^UNICEF, Dhaka, Bangladesh

**Keywords:** ready-to-use therapeutic food, severe acute malnutrition, Forcibly Displaced Myanmar Nationals, host communities, Bangladesh

## Abstract

**Introduction:**

There is a paucity of data on community perception and utilization of services for wasted children in Forcibly Displaced Myanmar Nationals (FDMN) and their nearest host communities.

**Methods:**

We conducted a qualitative study to explore community perceptions and understand the utilization of services for severely wasted children among the FDMN and their nearest host communities in Teknaf, Cox’s Bazar. We carried out 13 focus group discussions and 17 in-depth interviews with the caregivers of the children of 6–59 months, and 8 key informant interviews.

**Results:**

Caregivers’ perceived causes of severe wasting of their children included caregivers’ inattention, unhygienic practices, and inappropriate feeding practices. However, the context and settings of the FDMN camps shaped perceptions of the FDMN communities. Caregivers in both the FDMN and host communities sought care from healthcare providers for their children with severe acute malnutrition (SAM) when they were noticed and encouraged by their neighbors or community outreach workers, and when their SAM children suffered from diseases such as diarrhea and fever. Some caregivers perceived ready-to-use therapeutic food (RUTF) as a food to be shared and so they fed it to their non-SAM children.

**Discussion:**

Caregivers of the children having SAM with complications, in the FDMN and host communities, were reluctant to stay in stabilization centers or complex respectively, due to their households’ chores and husbands’ unwillingness to grant them to stay. The findings of this study are expected to be used to design interventions using locally produced RUTF for the management of SAM children in the FDMN, as well as to inform the health sector working on SAM child management in the host communities.

## Introduction

Utilization of healthcare services is an essential determinant of health (1) and has particular relevance as a public health and development issue in low-income countries (2). In fact, this utilization for the most vulnerable and underprivileged populations has been recommended by the World Health Organization (WHO) as a basic primary healthcare concept (3). Globally, one of the most vulnerable populations is the forcibly displaced population, and the number of this population has substantially increased, particularly in the last couple of years (4).

The recent Forcibly Displaced Myanmar National (FDMN) crisis in Southeast Asia has been alarming for humanity. Violence burst out in the late ‘70s in Myanmar, resulting so far in the displacement of about 1 million FDMN from its Rakhine state to neighboring Bangladesh (5). However, the largest influx of FDMN due to the recent immoral military operation of Myanmar has acutely worsened the situation (6). They are accommodated in a number of congested and resource-poor camps established by the Government of Bangladesh in Ukhiya and Teknaf Upazila of Cox’s Bazar district. As estimated in 2020, there were 160,544 children under 5 years of age in the total population (7). A study reports that under-5 children constitute 18.7% of the Rohingya diaspora community in Bangladesh, and 1.4% of children are wasted (8). Moderate wasting can result in a mortality rate of 30–148 per 1,000 children per year (9), and severe wasting is associated with a mortality rate of 73–187 per 1,000 children per year (10, 11). Therefore, healthcare services for wasted children are warranted.

Although the coverage of major humanitarian assistance programs such as household food rations and fortified blended foods has increased significantly (12), the concern is how the FDMN diaspora community is using the services provided by these programs. It has been suggested that healthcare should be universally accessible without barriers based on affordability, physical accessibility, or acceptability of services (2, 13). Unfamiliarity with healthcare services and traumatic migration experiences may pose challenges to seeking care and utilization of services for the forcibly displaced population. This situation can be worsened in the case of women and children.

Health seeking behavior is one of the key factors in utilizing services for wasted children like any healthcare services (2). The community uses services if they perceive them to be acceptable and beneficial. A good number of studies have been conducted to understand the perceptions and knowledge of the caregivers of under-5 children relating to facility-based healthcare services (14–18). However, most of the studies are from the African settings and very few of them have been carried out in the context of forcibly displaced populations. To our knowledge, none of the studies was done on caregivers’ perceptions and understanding of the utilization of services for severely acute malnourished (SAM) children among the FDMN and their nearest host communities. So, there is a paucity of data on the utilization of services for the SAM children in the FDMN camps and host communities, especially how they perceive the management of SAM children, particularly with ready-to-use therapeutic food (RUTF).

The WHO recommended a cut-off for weight-for-height of below −3 standard deviations or mid-upper arm circumference less than 115 millimeters to identify SAM in 6–59 months infants and young children (19). RUTF is used for the management of SAM children in the context of community-based management of acute malnutrition, and there has been speculation about whether RUTF should be produced primarily offshore, locally or both (20). Although offshore manufacturers offer RUTF at a lower cost, local production is approaching cost parity for RUTF. UNICEF, which purchases the majority of RUTF globally, continues to support local production, and efforts are underway to close the cost gap even further (20). The Government of Bangladesh has developed national guidelines for the management of SAM. However, policymakers and other stakeholders in Bangladesh are cautious about implementing community-based management of acute malnutrition program using RUTF due to concerns that relying on imported RUTF may not offer a sustainable solution.

icddr,b-an international public health research institute based in Dhaka, Bangladesh, with support from UNICEF and Nutriset developed two RUTFs termed as Sharnali1 and Sharnali-2 using locally available ingredients (21). Sharnali-1 was based on chickpeas and Sharnali-2 was based on rice-lentils. The study demonstrated that both the RUTFs were accepted by the children with SAM. However, the study was not conducted in a real emergency setting like FDMN camps where the number of SAM children were likely to be higher. Therefore, icddr,b with support from UNICEF initiated an effectiveness trial of Sharnali-1 and Sharnali-2 in the FDMN camps in Cox’s Bazar Bangladesh. Yet we did not know how the FDMN community would respond if we implemented such a service in their communities. Therefore, at the initial phase of the effectiveness trial, we conducted a qualitative study to explore community perception and understanding of access and utilization of services for the wasted children among the FDMN and their nearest host communities. We included the nearest host communities in the study to get a comparative understanding of the perceptions and utilization of services between the FDMN and host communities and what aspects especially need to be taken into consideration while designing the intervention for the effectiveness trial in the FDMN community.

The findings generated from this study helped us devise interventions to manage SAM in the FDMN camps. We anticipate that the evidence generated from this study will also inform the health sector on suitable, culturally acceptable, and feasible service delivery for SAM children among the FDMN population and their host communities.

## Materials and methods

### Study design

We used a qualitative approach to conduct the study. The study was exploratory in nature that gave us a better understanding of the community perception and the utilization of services for the wasted children among the FDMN and their nearest host communities of Cox’s Bazar, Bangladesh.

### Study participants

We included caregivers of children of 6–59 months. Caregivers were defined as mothers or other family members who took care of their children most of the time. They were identified by field staff who were aware of the local population and could identify households with a young child. We conducted focus group discussion with this group.

We also purposively selected the caregivers whose children had recently (within 6 months prior to data collection) received, or were receiving, inpatient or outpatient treatment for SAM in any healthcare facilities in study areas (camps and host communities). These caregivers were identified using hospital records and visiting households (for host communities) and the records in the Integrated Nutrition Facilities (INFs) in the camps. We conducted IDIs with this group.

We also selected some key informants such as Community Health Care Providers (CHCPs) from host communities, *Majhis* (community leaders) from the FDMN communities, Physicians, and outreach-and site-supervisors from the INFs in the camps. During the selection of key informants, we applied an intensity sampling procedure.

### Eligibility criteria

Mothers or caregivers who had children of 6–59 months and lived in the FDMN camps and their nearest host communities. Mothers or caregivers of those children who had recently received or were receiving inpatient or outpatient treatment for their SAM children.

For key informants, health service providers or managers of the healthcare facilities providing services for wasted and SAM children in the study area.

### Exclusion criteria

Mothers or caregivers of children of more than 5 years of age in the study area. The health service providers or managers of the healthcare facilities that did not provide any services for SAM children.

### Study site

The study was carried out at camps 25 and 27, and their nearest host communities in the Teknaf subdistrict of Cox’s Bazar district. The Government of Bangladesh has partnered with different non-governmental organizations and development agencies to form a nutrition sector (NS) and the NS is committed to providing nutritional services in the FDMN camps and host communities in Cox’s Bazar. The NS partners have been providing outpatient therapeutic treatment (OTP), targeted supplementary feeding program (TSFP), and blanket supplementary feeding program (BSFP) in the FDMN camps, and OTP and TSF programs in the host communities, including infant and young child feeding and other cross-cutting issues. As of March 2023, the partners of the NS were operating 45 INFs and five stabilization centers (SCs) or SAM corners, three SCs in the FDMN camps and two SAM corners in the host communities (22).

In the host community, the community clinic (CC) was the first formal contact of the caregivers for the SAM children, and if a child was found to be SAM with complication (s), she or he was referred to the nearest Upazila (sub-district) Health Complex (UHC). However, the CCs in the host communities were not like the CCs of the other general settings in Bangladesh because different international non-governmental organizations and national non-governmental organizations (NGOs) collaborated with the CCs to provide services for the SAM children.

The selection of study areas was based on the emergency prevailing in the area. Due to a nutritional emergency, the site was selected where, later, the intervention of an effectiveness trial of locally produced ready-to-use therapeutic food– Sharnali 1 and Sharnali 2 would be rolled out among the children of the FDMN suffering from SAM.

### Data collection

Data collection was conducted from March 2022 to August 2022. To achieve study objective, different data collection techniques were applied to collect data from different types of study participants. Four experienced Field Research Assistants (FRAs) under the supervision of an investigator (MR) were engaged in data collection and they had an academic background in Social Science. Two of them were females and the other two were males. They were recruited from the local areas so that they could speak and understand the dialect of the participants. Two investigators (MR and NNN) also conducted interviews with some key informants. The female Field Research Assistants approached the female participants (caregivers), and the male Field Research Assistants approached the male participants (*Majhis* and other male key informants) for interviews and focus group discussions. Before starting the interviews or focus group discussions the data collectors visited the households of the participants and built rapport with them. Thus, none of the study participants refused to participate in the study. They informed them about the goal and objectives of the study and took well-informed written consent from them before data collection. The FRAs noted down the issues pertaining to study objective and what they observed during data collection and study site visits and shared them with the investigators. The following data collection techniques were applied:

#### Focus group discussion

We conducted a total of 13 (6 from the host communities and 7 from the FDMN camps) Focus Group Discussions (FGDs) with the mothers or caregivers of the children of 6–59 months to understand their perceptions and practices on utilizing services for the wasted children. A trained moderator who was experienced in qualitative data collection facilitated the FGD sessions in the local language (dialect). A note taker also assisted the moderator in taking notes, probing for clarification and getting comprehensive narratives of the discussion topics. A guideline (Supplementary Table S1) was used to facilitate the discussion session in the context of the mother’s or caregiver’s beliefs, values, principles, priorities, barriers, gaps, and attitudes toward service utilization including perceived causes and consequences till, no important new themes were raised.

On average 8 mothers or caregivers of the children attended each FGD session from FDMN camps and 9 mothers or caregivers participated in each FGD session from host communities. Discussions were recorded on a digital recording device. The relevant and emerging key issues were noted down in a notebook by the moderator’s assistant. Each FGD took an average of 110 min in FDMN camps and 113 min in the host communities. The FGD sessions at the host communities were conducted at convenient places (the courtyard of the community or the house of a community member) considering the proximity to participants. The FGD sessions among the participants from FDMN were conducted at the INFs of the respected camps. Caregivers of the children who could not manage attendants to keep their children at home participated with their children in some of the FGDs.

#### In-depth interview

To get in-depth insights and understand caregivers’ experiences of receiving services for their wasted children, we conducted in-depth interviews (IDIs) with the purposively selected caregivers of the children who had recently received or were currently receiving, inpatient or outpatient treatment for SAM in INF (for FDMN) and any healthcare facilities in Teknaf, Cox’s Bazar (for host communities). The list of the children was collected from the facilities and/or identified by visiting the households of the caregivers of the children. The participants were purposively selected to maximize the variation in terms of their education, age, number of children etc. All the IDIs were conducted at the household level. A topic guideline (Supplementary Table S2) was used to conduct the interview. Each IDI lasted on average 86 min. We conducted a total of 17 IDIs (8 from the host communities and 9 from the FDMN communities) considering the principles of data saturation.

#### Key informant interview (KII)

We conducted key informant interviews with 2 *Majhis* (community leaders of the FDMN), 2 community health care providers at the host communities, 2 physicians, 1 site supervisor and 1 outreach supervisor from INFs to get insights into their experiences, provision of different services for the wasted children and suggestions on optimizing the services for the FDMN and their nearest host communities. On average 53 min was required to conduct a KII. A guideline (Supplementary Table S3) was followed to conduct the KIIs.

### Data analysis

After the FGDs and interviews, recordings were immediately transcribed/summarized by experienced qualitative researchers. The transcripts/summaries were read thoroughly for familiarity with the data. Peer briefings were done, and field notes were shared with the investigators by the data collectors to get feedback on what issues they needed to investigate more in-depth. The investigator provided feedback on those immediately so that the data collectors could investigate more in-depth on those issues from the next interviews or discussions. In this iterative way, initial analysis begun during data collection.

We also validated our study findings by member checking procedures. During the member checking procedure, our research team went back to some (2%) study participants (mostly caregivers and some key informants) with their transcripts and read out the transcripts to the respective participants and took their opinions if the transcripts were okay from their perspectives. Neither the FRAs nor the investigators were involved in selecting the participants for member checking. A Coordinator who was not involved in data collection, transcript preparation and data analysis, selected the participants for member checking. During member checking the research team members also made an effort to get clarity on the issues (if any) that needed further clarification.

Finally, we performed content analysis. Two Research Assistants identified the condensed meaning units from the textual data, tabulated them, cross-checked with each other and finalized the meaning units. Then the coding was done by them, patterns were identified and interpreted accordingly. The preidentified themes used to guide data analysis included caregivers’ perceptions of SAM, healthcare seeking behaviors and caregivers’ experiences with the treatment of their SAM children. The findings of the study have been presented using a consolidated criteria for reporting qualitative research (COREQ) checklist (23) (Supplementary Table S4).

## Results

During data collection, our research team attempted to capture community perceptions and understand access and utilization of services for the SAM children among the FDMN and their nearest host communities. For this, the team made an effort to maximize the variation in respondent selection from different types of study participants. Table 1 presents some basic characteristics of the study participants.

**Table 1:** Basic characteristics of the study participants.

Characteristics	Camp	Host community
*FGD participants: caregivers of the children of 6–59 months*		
No. of participants	56	58
Average age (in years)	25 (Range 18–40)	26 (Range 18–52)
Number of caregivers completed ≥5 years of schooling	08	33
Number of caregivers completed <5 years of schooling	48	25
*IDI participants: caregivers of severe wasted children*		
No. of participants	09	08
Average age (in years)	26 (Range 20–33)	28 (Range 19–40)
Number of caregivers completed ≥5 years of schooling	01	02
Number of caregivers completed <5 years of schooling	08	06
*Key informants: Majhi*		
No. of participant	02	–
Average years of schooling	10	–
Role as a Majhi (in years)	05	–
*Key informants: physician*		
No. of participant	01	01
Years of schooling	17	17
Years of experience	05	08
*Key informants: community health care provider*		
No. of participant	–	02
Average years of schooling	–	16
Average years of experience	–	11
*Key informants: Site and outreach supervisor (outpatient program in Rohingya refugee community)*		
No. of participant	02	–
Average years of schooling	17	–
Average years of experience	04	–

We have presented the findings of this study under different thematic areas including caregivers’ perceived cause of severe wasting of children, how the communities describe the severely wasted children, healthcare seeking behavior and practices of the caregivers of the wasted children, facilitators of and barriers to treatment of severely wasted children without complication (s) using ready-to-use therapeutic food (the existing ones used in camps) and challenges in inpatient treatment of the severely acute malnourished children.

### Phrases or terms used in describing the SAM children in the FDMN and host communities

During focus group discussions with the caregivers of the children of 6–59 months, we asked what phrases or terms they used in describing wasted children in their communities. We explored some common terms or phrases used in describing a wasted child in both the FDMN and host communities. The common terms or phrases included *lada* and *baraimmya* (ill). Among the FDMN community, the wasted children were also termed as *chuchi* (thin), *chiuinya putu* (thin boy/girl) *horaippa* (weak), *din din mojhe jawa* (becoming thinner day by day) and *kuchail* (wasted children make disturbances such as crying).

While describing a severely wasted child, a mother in an FGD said:

*“The child will disturb (kuchail). For example, the child will cry and the child will not want to stay in mother’s lap, and will not sleep properly.”*

In the host communities, the wasted children were also termed as *myara*, *hyangla*, *chikon* (thin), *gurani* (short and thin), *kushot* (having physical problem), *nitya roga* (having a recurrent physical problem) and *pettua* (thin but having large abdomen).

### Caregivers’ perceived causes of severe wasting of children in the FDMN and host communities

We explored caregivers’ perceived causes of severe wasting of the children in both the FDMN and host communities. Caregivers’ perceived causes of severe wasting of their children included caregivers’ inattention, unhygienic practices, and inappropriate feeding practices. Further investigation on the inattention of the caregivers revealed that early marriage made the mothers less caring as they could not perceive how to take care of their children when the mothers were adolescents. Sometimes households’ chores made the caregivers unable to take care of their children as perceived by some of the caregivers during the FGDs. Some of the caregivers also perceived those recurrent diseases such as diarrhea, fever and cold could cause severe wasting of the children. In an FGD, a woman from a host community stated that the elderly people in the community perceived that if the mothers got soaked in water, their children would catch a cold and become ill. As a result, their children became wasted. A woman in an FGD said:

*“The elderly (Murubbi) in the community advises us not to soak in water for a long period. If we are soaked in water for a long period, it can cause cold for our children and the children may become ill. Thus, these children can be wasted later.”*

Some of the caregivers added that the elderly people in the community also perceived that children might get wasted if they were fed any food impulsively. Among the elderly people in a host community, it persisted that if a child was fed thin fish, the child would be thin as well, as a caregiver mentioned in an FGD. Few of the caregivers perceived that it was mothers’ undernutrition that could cause severe wasting of their children. A participant of an FGD in a host community added that if there were a lack of nutrients in the breast milk of a mother it might cause severe wasting for her child.

In both the FDMN and host communities, domestic violence also came up as a perceived cause of wasting. A woman in an FGD mentioned:

*“Some men torture their wives. Then the women always feel anxiety. They do not take care of themselves and their children. They do not take meals in time and do not feed their children in time. Thus, the children become wasted.”*

Apart from domestic violence, extramarital affairs of the mothers of the children were cited as an indirect cause of child wasting in both communities. In an FGD, a woman mentioned that if a mother engaged in an extramarital affair she did not care for her family and did not take care of the child as well and thus, the child became wasted. However, in the FDMN communities, we found that some of their perceptions were shaped by the context and settings of the FDMN camps. In the FGDs in FDMN camps, some of the caregivers mentioned that sprawling condition of the camps might result in the wasting of the children. They added that lack of ventilation in the camps might cause wasting of their children. Few of them mentioned that their inability to feed the children according to their preferences might lead to wasting of their children. Some of them also said that it was Allah’s will if a child would be wasted or not. A caregiver in a FDMN camp said:

*“He is Allah who gives it and He also cures it (Allah diyer de, Allah’r usilay gom oy de).”*

Short birth intervals also came up as a perceived cause of severe wasting as some of the caregivers from the FDMN communities mentioned. They perceived that due to the short birth interval, a mother had more young children in a household and so the mother could not take care of the children.

### Healthcare seeking behavior and practices of the caregivers of the wasted children in the FDMN and host communities

In the in-depth interviews with the caregivers of the children who had SAM, the majority of them practiced home remedies in both the FDMN (6 out of 9) and host communities (6 out of 8). However, some (4 out of 9 in both communities) of them also sought care from informal healthcare providers before receiving treatment from formal healthcare providers. We found some caregivers who fed smashed rice with vegetables and hotchpotch (a dish made of rice and lentils cooked together with various spices) to their children and some caregivers fed commercial powdered milk to their wasted children. We found some caregivers who sought care from traditional healers (*Kabiraz or Baidya*) for their wasted children perceiving that their children were wasted because of spiritual reasons. The traditional healers gave *Jhar-fook* (charms and incantations), *Panipora* (holy water), *Telpora* (holy oil) and *Tabiz-koboz* (amulet) as healing substances to the caregivers. We found a mother of a child who performed incantation to get rid of wasting in her child. She said:

*“People (elderly in the community) told me that evil spirits possessed my child (bachchainton chummadion) and they were sucking blood of him and that’s why my child was getting wasted (As they suggested) … I drew an eyeball on a paper and then I took a handful of rice on the paper. Then I made a boat with the paper and swept the body of my child with that boat and finally I threw that away.”*

She performed this practice three times, as she added. We explored what triggered the caregivers of the SAM children to seek care from formal health service providers. In the FDMN communities, visits by the outreach workers and measurement of MUAC of the children by them triggered the caregivers to seek care from the INFs for their SAM children. However, in both the FDMN and host communities, the caregivers also sought care for their SAM children by being noticed and encouraged by their neighbors, and when their SAM children suffered from diseases such as diarrhea and fever.

The caregivers of the children in FDMN communities visited *Pushtikhana* (INF) or health-post, and in some cases (3 out of 9) even private clinics outside the camps, among the formal healthcare facilities. On the other hand, the caregivers of the children in host communities visited community clinics, upazila health complex and icddr,b hospital, Teknaf.

### Facilitators of and barriers to treatment of severely wasted children using ready-to-use therapeutic food (RUTF) in FDMN communities

In this study, we intended to explore the facilitators of and barriers to the treatment of severely wasted children with the existing RUTF that was being used in the INFs of the FDMN camps. The existing RUTF was known as *lalpushti* (“red nutrition” as its packaging was red) to the caregivers of the children. We found that most of the children liked *lalpushti*. The outreach workers or volunteers assigned by the INFs to screen the wasted children in the camps were acceptable to the communities and helpful as some of the caregivers reported. They were acceptable to the community people as they were recruited from their communities as mentioned by a site Supervisor of an INF and a *Majhi* in interviews.

The *Majhis* (FDMN community leaders) were found to be supportive in the management of severely wasted children with the use of *lalpushti*. In an interview, a head Majhi mentioned that before introducing *lalpushti* in the community for the treatment of severely wasted children, they were oriented. Not only the *Majhis* but also the Imams (religious leaders) and respected elderly people were oriented about the program as the *Majhi* added. In the last year, he attended at least 10 meetings in the INF as he mentioned. Being informed about the RUTF, they also discussed it with the community people as the community people listened to them. Upon being asked by a question what should be done if a locally produced RUTF was launched in the FDMN community, a *Majhi* said that they should be oriented first as it was done earlier when the *lalpushti* was launched. He said that earlier the community people did not want to trust this *lalpushti*. It took almost 3 to 4 years to make them understand the importance of *lalpushti*. They had to attend meetings in the INFs frequently and they were advised to make the caregivers understand the benefits of *lalpushti*, and accordingly, they did those to reduce the misconceptions regarding RUTF as a *Majhi* mentioned. He said:

*“Earlier they (community people) thought that if their children were fed this lalpushti, they would loss their Iman (trust on their own religious beliefs). They would be transformed to other religion … But gradually these misconceptions did not exist then.”*

Despite having an enabling environment in the FDMN community there were some barriers to using RUTF for the treatment of severely wasted children as we found. Since the children liked to eat *lalpushti*, some caregivers of the children requested the *Majhis* to advocate for them so that they could get *lalpushti* from INFs even after graduation from severe wasting as a Majhi said. Some of the caregivers perceived *lalpushti* as a food to be shared and so they fed it to their older children (non-SAM). Thus, the SAM children were likely to miss some sachets. We also found some caregivers who reported that they felt discouraged feeding *lalpushti* once their children had diarrhea after taking this.

### Facilitators of and barriers to treatment of severely wasted children using ready-to-use therapeutic food (RUTF) in host communities

Like the SAM children of the FDMN camps, the SAM children of the host communities also liked to take RUTF, as reported by the caregivers. However, the caregivers in the host communities had to collect RUTF from the CCs and some of them mentioned ‘distance from their houses to the CCs’ and ‘unavailability of suitable transportation’ as the barriers to collecting RUTF from the CCs. Some caregivers reported that while *tomtom* (a type of electric rickshaw) was a viable mode of transportation for visiting the CCs, it was not suitable (in terms of seating arrangement) for carrying their children. They also expressed concern about transportation costs.

A caregiver said:

*“Transportation is a major issue (for us). Taking kids (to the CC) by a tomtom is challenging. It's costly and scary …”*

### Referral mechanism and challenges in inpatient treatment of the severely acute malnourished children with complications in the FDMN communities

We found that the severely acute malnourished children with complications were referred from the INFs in the camps to the nearest SCs. We visited a SC that was run by a global humanitarian organization. The SC was known as *Dudhkhana* (a house of milk) to the caregivers in FDMN communities. In an interview with a Medical Officer in an SC, we found that the SC received on average 35–40 SAM children with complications in a month. The Medical Officer added that they received those children from the INFs of 13 camps.

Most of the caregivers of the SAM children with complications did not want to stay in SC, as stated by a Medical Officer. He further explained that most of the husbands of the caregivers did not want to allow their wives to stay in the SC. The husbands claimed that they did not have available household members to do household chores, so they did not want their wives to stay in the SC. In addition, it was difficult for the husbands and other family members to take care of other younger children in the households in the absence of the caregivers or mothers who stayed with the SAM children in the SC, as the key informant added. On the other hand, the SC did not allow males to stay at night, so, the female caregivers had to stay with the children at night. Non-compliance to treatment in the SC was also an issue, as a Medical Officer added. He said:

*“Suppose I have an emergency patient and I have gone to attend that (Perceiving difficulties to feed the child), … mothers or her older child takes this feed (F75) instead of feeding the admitted child.”*

### Referral mechanism and challenges in inpatient treatment of the severely acute malnourished children with complications in the host communities

Collaborating partner NGOs provided support in screening and referral of SAM children with complications from the CCs to UHCs. However, the caregivers of the children in the host communities were also reluctant to stay in UHCs due to similar problems such as households’ activities, lack of support to take care of other children and husbands’ disallowance as the study explored in the cases of staying in SCs for the caregivers in camps. In contrast to the transportation support (ambulance from INFs to SCs) in the camps, the caregivers of the children in host communities had to bear the transportation costs which discouraged them from seeking care from the UHCs. A CHCP said:

*“Most of the caregivers of the SAM children with complications are from the poor families. So, this is a challenge for them to bear transportation and other costs (accommodation and foods for other members who accompany the caregiver and child) at upazila.”*

### Suggestions from the study participants to optimize the services for severely wasted children in the FDMN and host communities

During data collection, we sought suggestions on optimizing services for severely wasted children from the perspectives of service receivers and service providers in the FDMN and host communities. The caregivers from the FDMN communities suggested distributing RUTF and providing other services (i.e., height and weight measurement and appetite test) from the respective blocks in the camp where the caregivers resided. It would save their time and help them avoid crowds in the *Pushticenter* (INF), as they mentioned. The service providers from the INF and SC suggested to have a provision of more counseling for the caregivers so that they could feed RUTF to their children maintaining hygiene (handwashing before feeding). One of the service providers from SC said that sometimes caregivers of the children complained of abdominal discomfort in their children (what they called diarrhea) after feeding RUTF. Therefore, counseling on water, sanitation, and hygiene (WASH) practices for the caregivers was needed, as he suggested.

Since ‘distance’ was mentioned as a barrier to seeking services for their SAM children, caregivers proposed that healthcare facilities be located near the host community to reduce their out-of-pocket expenditure in terms of transportation costs for treatment.

## Discussion

This qualitative study explored caregivers’ perceptions of severe wasting of their children of 6–59 months and understood healthcare seeking behavior and utilization of services in the FDMN and host communities. We found perceptions and misperceptions regarding the severe wasting of the children in both communities that correspond with the findings from some other studies (18, 24, 25), although the studies were not carried out in emergency settings. Positive perceptions should be nurtured but misperceptions should be changed because these misperceptions could be risk factors for child wasting (26).

Contextual factors influence caregivers’ misconceptions. Individuals attempt to understand their immediate environment in a variety of ways and develop subjective interpretations of their experiences (27). That is why, we found that in the FDMN community, some of the caregivers of the children mentioned lack of ventilation as a perceived cause of severe wasting of their children. The caregivers of the FDMN community lived in a sprawling condition of camp setting which led them to perceive this. However, such a perception is linked with the socio-economic and environmental causes of malnutrition which is reinforced by the findings of several studies (28).

Like another study conducted in India, our study also found that severe wasting is not perceived as a disease and the caregivers did seek care for their wasted children until they observed any visible clinical symptoms that triggered them to seek care (29). Thus, it can result in a delay in treatment seeking for severely wasted children and so, the role of community volunteers is crucial in the early detection of severely wasted children in the community, particularly in the FDMN community where the local dialect is vital for counseling the caregivers. Engagement of community leaders is also crucial for caregivers’ trust, acceptance and utilization of services (30). In our study, the community leaders themselves suggested engaging them in sensitizing the caregivers so that they can contribute to the advocacy for locally produced RUTF. Caregivers could be skeptical about using any new product for their children and might not trust outsiders. So, advocacy by the community leaders whom they trust could be an avenue to make the newly and locally produced RUTF acceptable to the caregivers.

RUTF is the key to the management of SAM without complications and countries’ endorsement and demand for using RUTF for the management of SAM are increasing over time (31). Although RUTF spread can be safely and easily manufactured in little or big amounts in most places across the world (32), its use and endorsement in many countries are limited. It is still not endorsed by many countries due to its unavailability in the countries and the cost of the imported RUTF (33). The study suggests that countries lacking the capacity to produce RUTF from locally available food ingredients can benefit from the production capacity of other countries in the region (33). However, our study focused more on the barriers to and facilitators of utilizing services for the management of SAM children from the perspectives of demand-side given that the RUTF was available in the humanitarian setting.

Caregiver’s perceptions of RUTF as a food to be shared correspond with the findings of several studies carried out in limited-resource settings (18, 34, 35). A study reported that the sharing of RUTF not only stemmed from social norms but also from food insecurity (18). Sharing of RUTF with siblings and other non-SAM children in the community is one of the major causes of non-compliance with RUTF and this finding corresponds with the evaluation results of a UNICEF program in Kenya as well (36), and it can result in relapse case of SAM as the study indicated. Several studies also demonstrated that the unintended consequences of sharing RUTF could result in delayed recovery of SAM children (34, 35, 37). Therefore, the local volunteer engaged in the community-based program should counsel the caregivers on children’s compliance with local RUTF and the importance of it during the enrolment of the SAM children in the program or intervention.

Non-compliance with treatment was not only found in out-patient treatment for severely wasted children but non-compliance issue was also found in some cases of severely wasted children with complications in SC. Our study revealed multifaceted problems contributing to caregivers’ indifference to the referral of their children to the SC and staying there with their children. A study shows that caregivers’ involvement in household activities impedes them from taking inpatient treatment for their children with severe wasting and complications from the facilities (38). Our study has further explored the underlying causes of the reluctance of caregivers to stay in the SC, especially in the refugee setting. The causes include husbands’ disallowing due to household chores and difficulty to take care of other younger children, and less caring adolescent mothers who are not even at childbearing age.

This qualitative study has explored a wide range of perceptions and behaviors of the FDMN and their nearest host communities pertaining to severe wasting and utilization of services for their children of 6–59 months. Findings generated from this study will help us devise the intervention of the effectiveness trial of locally produced RUTF for the management of SAM children in the FDMN. Based on the study findings, the interventions should have components like organizing advocacy meetings with the community leaders and engaging them in communication for behavior change before launching locally produced RUTF in the FDMN communities. This study suggests incorporating counseling into the intervention of the effectiveness trial for eliminating misperceptions and nurturing positive perceptions regarding the utilization of locally produced RUTF among caregivers. It also suggests recruiting the local volunteer or staff and using dialect (local terms) while counseling the caregivers.

The implementers and policymakers should take those perceptions and behaviors into account before devising the interventions or programs for the prevention and treatment of severely wasted children in emergency settings like FDMN camps and their nearest host communities.

### Strengths and limitations of the study

This is the first study that has explored community perceptions regarding severe wasting, and practices and utilization of services for the wasted children in the FDMN and their nearest host communities. The findings of this study have been validated by member checking procedure. The study had some limitations too. We did not assess the knowledge of the caregivers, so we could not conclude how correctly they could identify a SAM child. However, this study explored a wide range of perceptions and behaviors of the FDMN and their nearest host communities pertaining to severe wasting and utilization of services that are likely to provide an evidence base for designing or redesigning the interventions intended to manage severe wasting of the children of 6–59 months with locally produced RUTF in the similar humanitarian settings.

## Conclusion

This study explored a wide range of perceived causes of severe wasting among children in the FDMN and their nearest host communities. The study also identified several barriers and enablers for the management of severely wasted children in the context of FDMN and their host communities. Therefore, it is suggested to address the barriers and consider the enablers while designing a program or intervention for the management of severely wasted children using locally produced RUTF in similar emergency humanitarian settings.

## Data availability statement

The raw data supporting the conclusions of this article will be made available by the authors, without undue reservation.

## Ethics statement

The studies involving humans were approved by Institutional Review Board (IRB) of icddr,b (International Center for Diarrhoeal Disease Research, Bangladesh). The IRB of icddr,b consists of the Research Review Committee and Ethical Review Committee. The studies were conducted in accordance with the local legislation and institutional requirements. Written informed consent for participation in this study was provided by the participants’ legal guardians/next of kin.

## Author contributions

MR contributed to conceptualization of the study and drafted the manuscript. MR, NN, and AA analyzed data. TA acquired funding for this study. MI, MM, GK, AF, MS, PM, and TA reviewed and edited the manuscript. All authors contributed to the article and approved the submitted version.
