# Supplementary material for: Community perception and utilization of services for the severe wasted children aged 6–59 months in the Forcibly Displaced Myanmar Nationals and their nearest host communities in Bangladesh: a qualitative exploration
Source: Front Nutr. 2024 Feb 14;11:1235436. doi: 10.3389/fnut.2024.1235436 (PMC10899428; doi:10.3389/fnut.2024.1235436)
Supplement: Supplementary file 1 [file Table_1.DOCX]

**Supplementary Table 1: Focus Group Discussion guideline**

| **Domain 1: Identifying an acutely malnourished/severely wasted child and care-seeking behavior** | |
| --- | --- |
| Tell me how you do understand your child is healthy or malnourished |  |
| 1. If you were to describe if your child is healthy, how do you describe it? 2. If you were to describe if your child is malnourished, how do you describe it? |  |
| Are there any other words or phrases used by other caregivers in your community used to describe a healthy child?  Are there any other words or phrases used by other caregivers in your community used to describe a malnourished child?  *Probe*: How do other caregivers describe (including inner meaning of the words or phrases)?  *Note: Try to note type of caregiver (e.g., mothers-in-law, sister etc.)* |  |
| Tell me what you commonly do when you feel your child is malnourished?  Probe: Any home remedies? what is the first contact, second contact etc.? |  |
| What would you do if your child seems healthy by other family members or neighbors even if she/he has illness? |  |
| **Domain 2: Perceived causes of severe wasting in children** | |
| Tell me what do you think about causes of malnutrition. How does your child behave when he or she is sick?  *Probe: cause of malnutrition- parental, familial or societal issues; local superstitions, taboo, so on…*  *Notes: Looking for symptoms, emotions, and behaviors (e.g. presence of edema, fever, recurrent infection, diarrhea, weight loss, lack of appetite).* |  |
| Do you think inappropriate management, lack of accessibility or affordability are responsible for SAM? If you think so, how do you describe that. (Please ask each question separately) |  |
| Think back to the last time your child was ill with something like diarrhea, or a fever. Did you take your child to health care centre? If not, why?  (*Try to probe the age as well)*  *Probe: Do caregivers look for anything different if a child is ill?* |  |
| **Domain 3: Views and preferences on management of children with severe wasting** | |
| What are factors or reasons that mothers and other caregivers are not aware of the signs/symptom that we just discussed (*list them*)?  *Probe: For example, do mothers with less education or family support understand severe wasting differently?*  *Note: Try to find out presence of dominant family member.* |  |
| Do you think children should get proper and timely treatment regardless of gender? Is the frequency of seeking healthcare services same for both male and female child?  *Probe: Focus on gender, especially mother has a female child.* |  |
| How the decision is taken at the family level for the treatment of severe wasted child?  Probe: What do you do if other family members force you not to treat your severe wasted child?  Do you always listen to them? (Probe: why or why not)  If you do not listen to them, what influences make you to treat your child? |  |
| **Domain 4: Perceived risks and benefits of community-based treatment versus inpatient treatment** | |
|  | |
| What do you think about facility based and community-based treatment regarding severe wasting?  Note: Have you ever received any RUTF? (Please show the sample of RUTF)  If yes, did you find this helpful? Did you find any challenges or barriers receiving RUTF? Did your child take RUTF willingly? (Probe: If yes, why do you think so; if not, why do you think so?) (due to taste, flavor etc.) |  |
| Is there any risk if you treat your severe wasted child with community based management? |  |
| What are the risk or benefit treating your child in community? (Please ask the questions separately) |  |
| What are your views/opinions about community-based severe wasting management?  Probe: Have you ever heard community-based severe wasting management has negative/positive outcome or consequences?  Elaborate, if there is any. |  |
| (Explain about Sharnali 1 and Sharnali 2 first and then ask about their views if we implement Sharnali 1 and 2 in their community |  |
| **Domain 5: Community networks for supporting children with severe wasting** | |
| Tell me what do you think about the role of community workers and NGOs in supporting children with severe wasting management  Probe: *what do they think about health care referral system* |  |
| Tell me how influential and well-off members of the community could help caregivers, especially by providing financial or in-kind support to the family |  |
| **Domain 6: Health service factors as determinants of utilization** | |
| Tell me about availability of the health care services for your children in the community |  |
| Do you think you receive adequate services in proper time (Probe: Why or why not?) |  |
| Do you think the service you want to utilize is Affordable (Probe: Why or Why not?) |  |
| **Domain 7: Barriers and challenges to receive services for severe wasting in community** | |
| Tell me if you ever had faced any challenges during utilization of services for your severe wasted children (for user participants)  Tell me what you would think about the challenges that might come up during receiving therapeutic food. |  |
| Tell me the factors that you perceive as barriers for utilization of the services for the severe wasted children |  |
| Tell me what you think to overcome the challenges/barriers |  |
