# Supplementary material for: Community perception and utilization of services for the severe wasted children aged 6–59 months in the Forcibly Displaced Myanmar Nationals and their nearest host communities in Bangladesh: a qualitative exploration
Source: Front Nutr. 2024 Feb 14;11:1235436. doi: 10.3389/fnut.2024.1235436 (PMC10899428; doi:10.3389/fnut.2024.1235436)
Supplement: Supplementary file 2 [file Table_2.DOCX]

**Supplementary Table 2: In-depth Interview guideline**

| **Domain 1: Basic characteristics of the caregivers of the children who have received or are currently receiving services for their wasted children** | |
| --- | --- |
| characteristics of the caregiver and the children (age, sex, education, socio-economic status of the household, number of children etc.) |  |
| **Domain 2: Health seeking behavior** | |
| Please tell me what happed to the child? (Probe: why and when did you feel that you should seek care for the child) |  |
| What did you do when your child was sick?  (probe: (Probe: what triggered you to seek care, first contact, second contact etc., any nonformal care seeking before receiving care from the formal healthcare provider) |  |
| **Domain 3: Challenges of or barriers to utilization of services for wasted children** |  |
| Please tell us your experience about the services you have received for your child?  (probe: behavior and attitudes of the providers, how do you feel about the services? Why do you feel so?  Please tell us how accessible the service was? (probe: awareness of the services, distance etc.)  Please tell us how affordable the service was? (probe: cost, support from family and community etc)  If there were any challenges during seeking care for the severe wasted children (probe: what were those, how he/she did at that time etc.) |  |
| **Domain 4: Suggestions to make the services more available, accessible and affordable** |  |
| What do you suggest making the service more available? (Probe: why do you suggest so?)  What do you suggest making the service more accessible? (Probe: Why do you suggest so?  What do you suggest making the service more affordable? (Probe: Why do you suggest so?) |  |
