# Supplementary material for: Community perception and utilization of services for the severe wasted children aged 6–59 months in the Forcibly Displaced Myanmar Nationals and their nearest host communities in Bangladesh: a qualitative exploration
Source: Front Nutr. 2024 Feb 14;11:1235436. doi: 10.3389/fnut.2024.1235436 (PMC10899428; doi:10.3389/fnut.2024.1235436)
Supplement: Supplementary file 3 [file Table_3.DOCX]

**Supplementary Table 3: Key Informant Interview guideline**

| **Domain 1: Basic characteristics of the key informant** |  |
| --- | --- |
| (age, sex, education, training etc)  How long have you been involved in community-based management of severe wasted children/providing services for the wasted children?  (probe: When did you involve? How long have you been in providing services in this community etc)  How did you get involved in the management of severe wasted children in the facility/community? |  |
| **Domain 2: Role of the key informant** |  |
| Would you please tell us about your role in community-based management of severe wasted child?  (Probe: what are the activities you perform?)  Please tell me your experiences in providing the services at the facility/community (Probe: Who comes to you and why, at what stages, what do you do then, how do they react/response while receiving services etc.) |  |
| **Domain 3: Challenges faced by the providers and caregivers from the perspectives of the key informant** |  |
| Any challenges faced by the providers (Probe: What are those: from supply side: logistics, supplies, expertise etc)  Any challenges faced by the caregivers (probe from demand side: caregivers’ knowledge and attitudes, community’s perceptions, norms, beliefs etc.) |  |
| **Domain 4: Suggestions to improve management of severe wasting** |  |
| Would you please suggest us to improve management of severe wasting in the community (Probe: Why do you suggest so?)  How it can be implemented as you suggested? (Probe: logistics, supplies, human resources, cost, cultural acceptability of the program etc) |  |
