# Supplementary material for: Community perception and utilization of services for the severe wasted children aged 6–59 months in the Forcibly Displaced Myanmar Nationals and their nearest host communities in Bangladesh: a qualitative exploration
Source: Front Nutr. 2024 Feb 14;11:1235436. doi: 10.3389/fnut.2024.1235436 (PMC10899428; doi:10.3389/fnut.2024.1235436)
Supplement: Supplementary file 4 [file Table_4.docx]

**Supplementary Table4: COREQ checklist**

| **No. Item** | **Guide questions/description** | **Reported on Page #** |
| --- | --- | --- |
| **Domain 1: Research team and reﬂexivity** |  |  |
| *Personal Characteristics* |  |  |
| 1. Inter viewer/facilitator | Which author/s conducted the interview or focus group? |  |
| 2. Credentials | What were the researcher’s credentials? E.g. PhD, MD |  |
| 3. Occupation | What was their occupation at the time of the study? |  |
| 4. Gender | Was the researcher male or female? |  |
| 5. Experience and training | What experience or training did the researcher have? |  |
| *Relationship with participants* |  |  |
| 6. Relationship established | Was a relationship established prior to study commencement? |  |
| 7. Participant knowledge of the interviewer | What did the participants know about the researcher? e.g. personal goals, reasons for doing the research |  |
| 8. Interviewer characteristics | What characteristics were reported about the inter viewer/facilitator? e.g. Bias, assumptions, reasons and interests in the research topic |  |
